# Determining gestational age and preterm birth in rural Guatemala: A comparison of methods

**DOI:** 10.1371/journal.pone.0193666

**Published:** 2018-03-19

**Authors:** John R. Weinstein, Lisa M. Thompson, Anaité Díaz Artiga, Joe P. Bryan, William E. Arriaga, Saad B. Omer, John P. McCracken

**Affiliations:** 1 School of Nursing, University of California, San Francisco, San Francisco, California, United States of America; 2 Centro de Estudios en Salud, Universidad del Valle de Guatemala, Guatemala City, Guatemala; 3 Central American Regional Office, Centers for Disease Control and Prevention, Guatemala City, Guatemala; 4 Division of Global Health Protection, Centers for Disease Control and Prevention, Atlanta, Georgia; 5 Ministerio de Salud Pública y Asistencia Social, Quetzaltenango, Guatemala; 6 Rollins School of Public Health, Emory University, Atlanta, Georgia, United States of America; Univesity of Iowa, UNITED STATES

## Abstract

**Background:**

Preterm birth is the leading cause of death among children <5 years of age. Accurate determination of prematurity is necessary to provide appropriate neonatal care and guide preventive measures. To estimate the most accurate method to identify infants at risk for adverse outcomes, we assessed the validity of two widely available methods—last menstrual period (LMP) and the New Ballard (NB) neonatal assessment—against ultrasound in determining gestational age and preterm birth in highland Guatemala.

**Methods:**

Pregnant women (n = 188) were recruited with a gestational age <20 weeks and followed until delivery. Ultrasound was performed by trained physicians and LMP was collected during recruitment. NB was performed on infants within 96 hours of birth by trained study nurses. LMP and NB accuracy at determining gestational age and identifying prematurity was assessed by comparing them to ultrasound.

**Results:**

By ultrasound, infant mean gestational age at birth was 38.3 weeks (SD = 1.6) with 16% born at less than 37 gestation. LMP was more accurate than NB (mean difference of +0.13 weeks for LMP and +0.61 weeks for NB). However, LMP and NB estimates had low agreement with ultrasound-determined gestational age (Lin’s concordance<0.48 for both methods) and preterm birth (κ<0.29 for both methods). By LMP, 18% were judged premature compared with 6% by NB. LMP underestimated gestational age among women presenting later to prenatal care (0.18 weeks for each additional week). Gestational age for preterm infants was overestimated by nearly one week using LMP and nearly two weeks using NB. New Ballard neuromuscular measurements were more predictive of preterm birth than those measuring physical criteria.

**Conclusion:**

In an indigenous population in highland Guatemala, LMP overestimated prematurity by 2% and NB underestimated prematurity by 10% compared with ultrasound estimates. New, simple and accurate methods are needed to identify preterm birth in resource-limited settings worldwide.

## Introduction

Worldwide, nearly fifteen million infants (11%) are born preterm each year [1]. Preterm birth is the 3^rd^ leading cause of Disability Adjusted Life Years (DALYs) across all age groups and is the leading cause of death in children <5 years of age, responsible for over 800,000 deaths per year (14% of total) [2]. This burden lies primarily in low and lower-middle income countries [2]. Childhood mortality is a major contributor to the overall human health burden and is a threat to health security. Determining the causes of childhood mortality is essential for developing interventions to prevent childhood mortality and enhancing global health security.

In Guatemala, there are an estimated thirty-six thousand preterm births (8%) per year [1], which are responsible for nearly 1,300 deaths [2]. Identifying infants born preterm is essential to prioritize care among those at highest risk [3]. Even among infants born term, those born at 37 and 38 weeks have markedly greater mortality [4] and lower academic achievement [5] than those born at 39 and 40 weeks. Additionally, many countries lack reliable preterm birth data [6] and, thus, reliable methods of detection are necessary to strength the availability and quality of the data.

Ultrasound during the first trimester of pregnancy is considered the most accurate method for determining gestational age [7]. However, this requires early identification of pregnancy, access to medical care, skilled ultrasound technicians, expensive equipment and maintenance. Furthermore, accuracy drops with increasing gestational age [8]. In Guatemala, less than 20% of women receive skilled antenatal care in the first trimester; only 31% of women have at least one skilled antenatal care visit with one third presenting after the first trimester [9]. Thus, given the need for early access to ultrasound for precise gestational age determination, other accurate methods–ones more feasible for use in low-resource settings, such as Guatemala where women typically present late to prenatal care—are desired.

Using the known date of last menstrual period (LMP) can be highly accurate. Estimates have been shown to be within days of those from ultrasound, even in low- and middle-income countries [10–16]. Additionally, there is fairly good agreement with ultrasound at identifying preterm births (κ > 0.72) [10, 11, 16] with a sensitivity and specificity above 85 and 95%, respectively [10]. The regularity of the female menstrual period, timing of ovulation and fertilization, mid-cycle bleeding, and oral contraceptives can affect accuracy [7]. Additionally, LMP is subject to recall bias and number preference (e.g. preference for 1^st^ or 15^th^ of month, or rounding to zero or five) [7, 16] and there is a systematic bias of less accurate recall of LMP among some subgroups, such as those with lower educational achievement [7, 11]. As such, estimates of gestational age from LMP can suffer from a lack of precision with some individual estimates differing by weeks compared with ultrasound estimates [10, 12, 13, 15].

Other methods for determining gestational age use neonatal physical and neuromuscular measurements to determine developmental maturity. These include the 21-item Dubowitz [17], 12-item Ballard [18, 19], the 6-item Capurro [20], and the 6-item Eregie [21]. Both the Capurro and Ballard have been shown to be skillfully performed in rural Guatemala by traditional birth attendants and nurses [13, 22]. Gestational age estimates from these methods can be accurate within several days [13, 15, 23, 24] and correlate quite strongly with other methods [15, 24–26]. However, similar to LMP, they can be imprecise [13, 23, 24] and, in general, overestimate gestational age [7]. Additionally, while they have been shown to have high specificity for determining preterm birth they suffer from low sensitivity [14, 23].

The purpose of this exploratory study is to compare gestational age and preterm estimates from ultrasound, LMP and Ballard under real field conditions and investigate factors influencing accuracy to determine the best method for use in the highlands of rural Guatemala.

## Materials and methods

### Recruitment and eligibility

This methodological study was part of a larger prospective cohort study completed between 2013 and 2015, the *Embarazo Seguro Bebé Sano* (Safe Pregnancy Healthy Baby) Study, investigating several potential environmental, nutritional and infectious risk factors for disease during pregnancy and infancy [27, 28]. Study participants were recruited from pregnant women presenting to Hospital Regional de Occidente—San Juan de Dios, the public primary care clinics in San Juan Ostuncalco or Concepción Chiquirichapa, and the clinics of the non-governmental organizations *Asociación de Comadronas del Área Mam* and *Pies del Occidente* from May 2013 to February 2014. Medical chart reviews and interviews of the pregnant women were used to determine eligibility. Women were eligible if they were non-smokers between the ages of 18 and 40 years old, had a low-risk, singleton pregnancy less than 20 weeks gestation by LMP or ultrasound and not greater than 24 weeks by the other method, lived and planned to stay in the area for at least six months post-delivery and had access to a mobile phone. Low risk pregnancies were defined as maternal blood pressure less than139/89 mmHg and no protein, glucose or ketones in the urine at the baseline clinical exam.

### Prenatal and newborn assessment

A home visit was made within one week after enrollment to assess baseline household and maternal characteristics. Weekly phone calls and cell phone text messages were conducted after baseline to assess pregnancy status until the mother delivered her infant. Mothers were instructed to contact the study personnel if they went into labor. Study personnel assessed newborns either at the hospital or in the home, depending on place of delivery and timing of the assessment.

### Determination of gestational age at birth

Three methods were used to calculate the gestational age at birth: fetal ultrasound, New Ballard and LMP. Preterm births were those infants having a gestational age at birth less than 37 weeks. As defined by the WHO, moderate to late preterm births are those between 32 and 37 weeks, very preterm between 28 and 32 weeks, and extremely preterm less than 28 weeks [29].

#### Last menstrual period

LMP was self-reported during the baseline clinical exam. If the exact date of the month could not be recalled, the 15^th^ day of the month was used. We determined the estimated delivery date (LMP + 280 days). The difference between the estimated delivery date by LMP and actual delivery date was then used to determine gestational age in weeks:

$$(40\;weeks-\frac{LMP\;estimated\;delivery-actual\;delivery}{7}).$$

#### New Ballard maturational assessment

Project nurses assessed newborn gestational age using the New Ballard (NB) maturational assessment. The New Ballard Score measures 6 physical and 6 neuromuscular criteria; the total score ranges from -10 to 50. Nurses were trained over 2 days; didactic training including videos of the New Ballard assessment was followed by hands-on assessments of newborns in the postpartum ward of the hospital.

NB scores were calculated for infants examined within 96 hours of birth in 156 (83%) of the live births. Typically the New Ballard gives gestational age in 2 week increments. To provide gestational ages in smaller increments, gestational age (in weeks) was determined from the NB score by a regression equation from published scores and estimated gestational ages (0.4*NB score + 24) [18].

#### Ultrasound

All mothers had an ultrasound taken as part of the screening for recruitment eligibility. To standardize measures among those performing ultrasounds, study physicians received didactic and hands-on training on ultrasound use before the study began, complemented by follow-up in-person trainings during the course of the study. Consistent quality measurements were required before physicians were permitted to perform ultrasounds for the study. For quality control, 33% of study images were assessed by a board-certified obstetrician and ultrasounds were repeated if they were found to be of low quality. When multiple ultrasounds were performed, the earliest set of high quality images was used. Before 14 weeks gestational age, crown-rump length was used to determine gestational age [30]. After 14 weeks gestation, gestational age was taken as the mean age determinations from biparietal diameter measurement, head circumference, femur length and abdominal circumference. If gestational age computed from any individual measure differed from the mean gestational age by more than the level of accuracy at that gestational age (1 week for ultrasounds obtained before 21 weeks or 2 weeks for ultrasounds obtained at 21 weeks or later) [8], that measure was excluded from the mean estimated gestational age. Additionally, all available images were evaluated to verify poor caliper placement used during fetal measurement and those with improper caliper placements were excluded from analysis. The difference between estimated delivery date by ultrasound and the actual delivery date was used to calculate gestational age at birth in weeks.

### Newborn anthropometrics

For the calculation of small for gestational age, newborn weight was measured to the nearest 5 grams using a Seca 334 digital infant scale. Weight was measured in triplicate and clothing weight was subtracted from infant weight. Measurements within 50 g of each other were used to determine the average. If either the birthweight or clothing weight were implausible (<1400 grams or >5000 grams for birthweight and <100 g or >1000 g for clothing) or taken later than two weeks after birth, the recorded birthweight from the hospital medical record was used, if available (n = 25), or excluded from analysis, if unavailable. Only weights taken within 2 weeks of birth (n = 184) were used in the estimation of small-for-gestational age.

### Statistical methods

All statistical analyses were performed in Stata 13. Descriptive statistics (proportion, mean, standard deviation, median, interquartile range) were used to describe the data.

Lin’s concordance correlations [31] and Bland-Altman plots [32] were used to determine agreement between different methods of measuring gestational age. Lin’s concordance correlation is the product of the Pearson correlation coefficient and a bias correction factor, a measure of accuracy that indicates how close the best-fit line deviates from the line y = x or perfect agreement between methods. Fixed biases (systematic differences that can be adjusted for in analyses), or mean difference between methods, were assessed using the Bland-Altman plots. In addition, Bland-Altman plots were used to assess the 95% limits of agreement or the range in which 95% of differences would be expected to fall. Spearman rank correlation was used to determine correlation between ultrasound-determined gestational age and individual New Ballard item scores or physical and neuromuscular subscores. Multivariate linear regression was used to assess the association of maternal and infant characteristics on the difference in gestational age estimates compared to ultrasound. Differences in these differences based on sociodemographic characteristics were estimated from a linear regression equation using maternal and infant characteristics as explanatory variables.

Cohen’s kappa statistic [33] was used to assess agreement in the determination of preterm birth between the different methods of measuring gestational age. Using ultrasound as the gold-standard method, the sensitivities, specificities, and diagnostic odds ratios [34] for each method were determined. The diagnostic odds ratio (DOR) is the ratio of the odds that the method identifies a preterm infant as preterm to the odds that it identifies a full-term infant as preterm. Higher DORs are indicative of better performance and a DOR of 1 indicates that the test is as likely to predict that an infant is preterm regardless of whether they are. Associations between NB item scores and preterm birth were determined by Fisher’s exact test and differences in physical and neuromuscular subscores between full and preterm birth were tested for statistical significance using the Wilcoxon rank sum test.

Small-for-gestational age was defined as an infant being below the 10^th^ weight centile for gestational age as determined by the INTERGROWTH-21st Newborn Size Application Tool using standards derived from births in eight ethnically distinct countries (http://intergrowth21.ndog.ox.ac.uk/).

### Ethical approval

This study was approved by human subjects committees at Universidad del Valle de Guatemala, Emory University, and the Guatemalan Ministry of Public Health and Social Welfare. This study was reviewed in accordance with CDC human subjects protection policy and was determined to be human subjects research, but CDC involvement did not constitute engagement in human subjects research. All participants signed a written consent that was read to them in Spanish and, if they were unable to read or write, a witness also signed to ensure comprehension by the participant.

## Results

**Fig 1:**
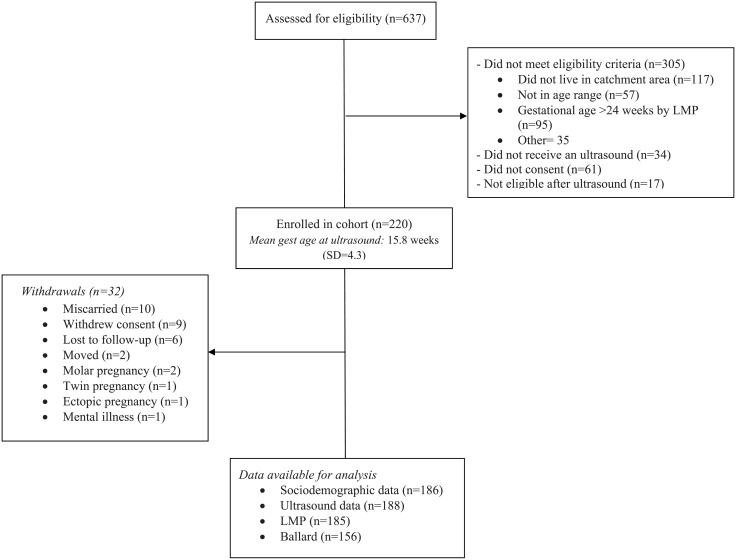
Recruitment and follow-up flow diagram.

In total, 637 women were assessed for eligibility with 221 meeting all inclusion criteria and consenting to participate in the study (Fig 1). The mean ultrasound-determined gestational age at recruitment among the eligible cohort was 15.8 weeks (standard deviation (S.D.) = 4.3). Among those women followed to birth (N = 188), mean ultrasound-determined gestational age at recruitment was 16.1 weeks (S.D. = 3.7) with 23% having an ultrasound in the first trimester of pregnancy (<13 weeks gestational age). Among women who had an ultrasound in the second trimester (N = 145; Table 1), 27 had one between 20 and 24 weeks gestational age, of which 10 were repeats due to inaccurate first readings. The median age among the study women was 23 years old (interquartile range (IQR): 20–30). The majority spoke exclusively Mam at home (76%), had a monthly household income < $133 (69%), and had a primary school education or less (68%). Preterm birth occurred in 16% (95% CI: 11–22%) of pregnancies based on ultrasound dating and 31% (95% CI: 25–38%) of infants were born small-for-gestational age. Only one birth was considered very preterm with the rest being moderate to late preterm.

**Table 1:** Sociodemographic characteristics of study participants.

	No. of pregnant women (N = 188)	%
*Language spoken at home*:		
Spanish spoken at home	44	24
Mam only	142	76
*Monthly income*:		
<$133	128	69
$133–398	43	23
Don’t know/no response	15	8
*Education*:		
No formal school	19	10
Primary	107	58
Middle-school	30	16
High school or greater	30	16
Age, years (median (IQR))	23 (20–30)	
Gestational age at ultrasound		
Weeks, mean (SD)	16.1 (3.7)	
First trimester	43	23
Second trimester	145	77
*Recall of LMP*		
Recalls exact date	154	82
Recalls month, but not day	34	18
Preterm birth (by ultrasound)	30	16 (95% CI: 11–22)
Moderate to late preterm (32–37 weeks)	29	
Very preterm (28–32 weeks)	1	
Extremely preterm (<28 weeks)	0	
Small-for-gestational age	56	31 (95% CI: 25–38)

### New Ballard compared to ultrasound

**Fig 2:**
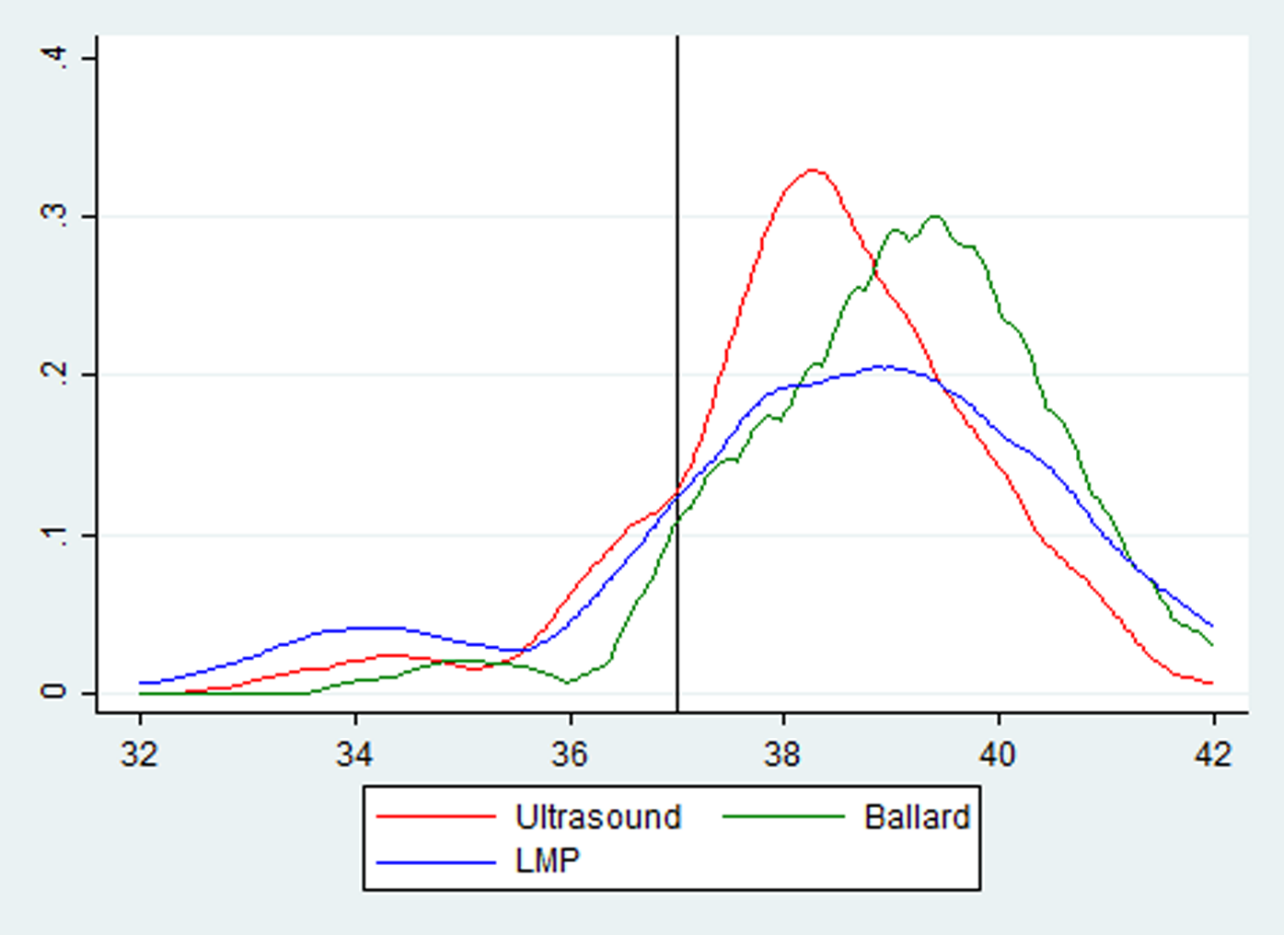
Gestational age distributions by NB, LMP and ultrasound. The vertical line indicates 37 weeks, the threshold between term and preterm births (y-axis is the kernel density of the gestational age distribution for each method).

Overall, the distribution of gestational ages as determined by NB was shifted toward higher gestational ages compared to ultrasound (Fig 2) by an average of 0.61 weeks (Table 2). However, there was only modest agreement between individual gestational age estimations using the New Ballard and ultrasound with a Pearson’s correlation of 0.42, a bias correction factor of 0.92 and Lin’s concordance correlation coefficient of 0.39 (Table 2). Additionally, only 42% and 74% of Ballard gestational age estimates were within one week and two weeks of gestational age based on ultrasound, respectively (S1 Table). The 95% limits of agreement between the New Ballard and ultrasound—the range in which 95% of the differences between the methods would be expected to lie—was from -2.51 weeks to 3.73 (Table 2 and S1A Fig) indicating wide variation in accuracy of individual estimates. These differences in gestational age estimates by New Ballard led to clinically relevant differences in estimates on small for gestational age (SGA); using New Ballard to determine gestational would have overestimated the proportion of SGA infants by over 10% compared with ultrasound dating (45% versus 34%; Table 3) among those infants on which New Ballard was performed and birthweight was known (N = 155).

**Table 2:** Performance of LMP and Ballard to determine gestational age at birth compared to ultrasound.

	Gestational age at birth						Preterm birth						
	Mean in weeks (SD)	Convergent validity			Bland-Altman statistics		Proportion preterm	kappa	Sensitivity	Specificity	Diagnostic Odds Ratio (95% CI)	Positive Predictive Value	Negative Predictive Value
		Pearson’s correlation coefficient	Bias Correction Factor	Lin’s concordance correlation (95% CI)	95% limits of agreement, weeks	Mean difference, weeks (CI)							
**Ultrasound (n = 188)**	38.3 (1.6)	(Ref.)	(Ref.)	(Ref.)	(Ref.)	(Ref.)	16.0%	(Ref.)	(Ref.)	(Ref.)	(Ref.)	(Ref.)	(Ref.)
**New Ballard (n = 156)**	39.0 (1.4)	0.42	0.92	0.39 (0.26, 0.51)	-2.51 to 3.73	0.61 (0.36, 0.86)	6.4%	0.1090	13.6%	94.8%	2.61 (0.73, 9.34)	30.0%	87.0%
**LMP (n = 185)**	38.4 (2.3)	0.52	0.92	0.48 (0.38, 0.58)	-3.93 to 4.20	0.13 (-0.16, 0.43)	17.8%	0.2925	43.3%	87.1%	5.16 (2.21, 12.09)	39.4%	88.8%

**Table 3:** Estimates of proportion of small-for-gestational age (SGA) infants by LMP and Ballard compared to ultrasound.

	Proportion SGA by method (95%CI)	Proportion SGA by ultrasound (95%CI)
New Ballard (n = 155)	45% (37–52)	34% (27–42)
LMP (n = 176)	38% (31–46)	32% (25–39)

The accuracy of gestational age estimates by the New Ballard was dependent on trimester of ultrasound and whether the infant was preterm or SGA. The mean difference in gestational age estimates between New Ballard and ultrasound was larger among those having a first trimester ultrasound, when ultrasonography is most accurate, compared to those with second trimester ultrasounds (+1.10 weeks vs. +0.47 weeks in second trimester) and among preterm infants (Table 4). New Ballard was far more accurate among SGA infants based on ultrasound; the mean difference in gestational age estimates among SGA infants was less than a day (+0.05 weeks), compared to +0.88 weeks for non-SGA infants (Table 4).

**Table 4:** Difference between ultrasound and LMP or New Ballard-determined gestational age by maternal and infant characteristics.

	Gestational age difference (New Ballard-US), weeks^a^		Gestational age difference (LMP-US), weeks	
	Mean difference (sd)	Difference of the difference^b^ (95% CI)	Mean difference (sd)	Difference of the difference^b^ (95% CI)
*Gestational age at ultrasound (per extra week)*			-	-0.18 (-0.26, -0.10)
*Recall*				
Exact date			0.11 (1.90)	Reference
Month only			0.24 (2.62)	0.46 (-0.29, 1.21)
*Years of school*				
Primary or less			0.12 (2.1)	Reference
Greater than primary			0.18 (1.9)	0.01 (-0.63, 0.65)
*Age*				
≤23 years			0.20 (2.16)	Reference
> 23 years			0.07 (1.89)	-0.18 (-0.77, 0.41)
*Ethnicity*				
Spanish speaking	0.40 (1.65)	Reference	0.43 (1.84)	Reference
Mam only	0.67 (1.54)	0.32 (-0.21, 0.85)	0.06 (2.09)	-0.41 (-1.07, 0.26)
*Gender*				
Male	0.88 (1.54)	Reference	-0.21 (2.01)	Reference
Female	0.38 (1.55)	-0.32 (-0.77, 0.14)	0.43 (2.01)	0.46 (-0.11, 1.03)
*Term status*				
Full-term	0.39 (1.48)	Reference	-0.02 (1.87)	Reference
Preterm	1.97 (1.40)	1.52 (0.87, 2.17)	0.94 (2.61)	0.98 (0.19, 1.77)
*SGA*				
Non-SGA	0.88 (1.54)	Reference	0.26 (2.16)	Reference
SGA	0.05 (1.47)	-0.83 (-1.31, -0.34)	-0.10 (1.56)	-0.18 (-0.70, 0.43)

^a^ Gestational age at ultrasound, recall of the LMP, and maternal age and education were assumed to be independent of the accuracy of the nurse-administered New Ballard and thus were not included for analysis

^b^ Difference of the difference holding all other variables constant calculated as the β from multivariate linear regression using all maternal and infant characteristics as explanatory variables

### LMP compared to ultrasound

LMP performed better than the New Ballard at determining gestational age. While the distribution of gestational age estimates by LMP was more disperse (Fig 2), it overestimated gestational age by a mean 0.13 weeks (Table 2), less than the mean difference with the New Ballard. Additionally, more gestational estimates by LMP were within two weeks of ultrasound dating than with NB; 56% of LMP gestational age estimates were within one week and 79% were within two weeks (S1 Table). This was despite 18% of women being able to recall only the month of their last menstrual period (Table 1). There was also stronger agreement in individual estimates between these two methods (ultrasound and LMP) than between ultrasound and New Ballard with higher Pearson’s (0.52) and Lin’s concordance correlation coefficients (0.48; Table 2). The limits of agreement between LMP and ultrasound (-3.93 to 4.20 weeks) were higher than those between the New Ballard and ultrasound, however (S1B Fig), indicating poorer precision with LMP than Ballard. Additionally, agreement differed by trimester of ultrasound with a higher Lin’s concordance correlation between LMP and ultrasound-determined gestational age among those with a second trimester ultrasound (correlation coefficient = 0.55 vs 0.20 for first trimester ultrasounds). Similar to NB, determining gestational with LMP overestimated the proportion of infants born small for gestational age compared with ultrasound (38% vs. 32%) among those pregnancies in which an LMP and birthweight were known (N = 176) (Table 3).

The accuracy of LMP estimates differed by gestational age at ultrasound and whether the infant was preterm. The gestational age of infants born preterm according to ultrasound were overestimated by LMP by one week compared with term infants (Table 4). There was also a strong and significant trend in difference between gestational age at birth based on gestational age of ultrasound (β = -0.18; Table 4); for ultrasounds performed early, LMP overestimated gestational age at birth (1.3 weeks among infants having 1^st^ trimester ultrasounds) but underestimated gestational age at birth for later ultrasounds. Mother’s age and educational attainment had no statistically significant effect on the accuracy of the LMP estimation (Table 4).

### Determination of preterm birth

New Ballard greatly underestimated the rate of preterm birth (6.4%) in the study population, whereas LMP slightly overestimated preterm birth (17.8%) compared to ultrasound (16.0%) (Table 2). LMP had a larger kappa statistic (0.29 vs 0.11) than the New Ballard, however, this represented only low to moderate agreement with ultrasound (Table 2). While LMP performed better, both tests suffered from low sensitivity (New Ballard: 15.8% and LMP: 43.3%), moderate to high specificity (New Ballard: 93.3% and LMP: 87.1%) and low diagnostic odds ratios (New Ballard: 2.60 and LMP: 5.16; Table 2). The 95% confidence interval of the diagnostic ratio for the New Ballard crosses 1 indicating no significant discriminative ability at discriminating preterm from full-term infants. A determination of preterm birth by LMP alone would be incorrect 60.6% of the time (positive predictive value of 39.4%) and that of full-term would be incorrect 11.2% of the time (negative predictive value 88.8%) (Table 2). After adjusting Ballard by its fixed bias, it performed similarly to LMP at determining whether a birth was preterm or not.

### Association of individual New Ballard item scores with gestational age and preterm birth

Because the New Ballard is a composite of six neurological and six physical criteria, we investigated whether some measures of the New Ballard scale performed better than others. While the physical and neuromuscular subscores had similar correlations with gestational age, more individual neuromuscular (4/6) than physical scores (3/6) were significantly correlated with ultrasound-determined gestational age (Table 5). Similarly, some New Ballard measures were more predictive of preterm birth than others. Both physical and neuromuscular subscores of preterm infants (as determined by ultrasound) were significantly different than those born at term (Table 5), indicating the ability to distinguish newborn characteristics between preterm and full-term infants. However, individual neuromuscular scores were more strongly associated with preterm birth; 5 of 6 neuromuscular scores had significant associations with preterm birth whereas the relationship was significant for only one of the physical scores, breasts (Table 5).

**Table 5:** New Ballard scores and correlation to ultrasound-determined gestational age and preterm birth.

	Mean score (SD)	Mean score of full-term (SD)	Mean score of preterm (SD)	Correlation with gestational age (ρ)
Skin	2.84 (0.65)	2.87 (0.64)	2.68 (0.72)	0.23***
Lanugo	1.97 (0.80)	2.00 (0.81)	1.82 (0.78)	0.10
Plantar Surface	3.63 (0.54)	3.65 (0.54)	3.50 (0.51)	0.13*
Breasts	3.04 (0.80)	3.13 (0.78)	2.55 (0.74) ^ŦŦŦ^	0.27***
Eye/Ear	3.06 (0.50)	3.06 (0.52)	3.05 (0.38)	0.15*
Genitals	2.89 (0.67)	2.94 (0.66)	2.59 (0.67)	0.29***
*Physical Maturity Subscore*	17.44 (2.06)	17.65 (2.05)	16.18 (1.68)^^^^^	0.35***
Posture	3.49 (0.55)	3.54 (0.54)	3.23 (0.53) ^ŦŦŦ^	0.15*
Square Window	3.67 (0.51)	3.72 (0.48)	3.32 (0.57) ^ŦŦŦ^	0.28***
Arm Recoil	3.46 (0.62)	3.51 (0.57)	3.14 (0.77) ^ŦŦŦ^	0.29***
Popliteal Angle	3.41 (0.64)	3.46 (0.66)	3.09 (0.43) ^ŦŦŦ^	0.04
Scarf sign	2.92 (0.57)	2.93 (0.58)	2.86 (0.56)	0.20**
Heel to Ear	3.08 (0.65)	3.14 (0.58)	2.68 (0.89) ^ŦŦŦ^	0.23***
*Neuromuscular Maturity Subscore*	20.02 (2.00)	20.31 (1.89)	18.32 (1.86)^^^^^	0.33***

* = p<0.1,

** = p<0.05, and

*** = p<0.01 for Spearman correlation between Ballard score and ultrasound-determined gestational age at birth.

^Ŧ^ = p<0.1,

^ŦŦ^ = p< 0.05, and

^ŦŦŦ^ = p < 0.01 for association between Ballard score and preterm birth by Fisher’s Exact test.

^ = p<0.1,

^^ = p< 0.05, and

^^^ = p< 0.01 for difference within subscore between full-term and preterm infants by the Wilcoxon Rank Sum test.

## Discussion

LMP performed better than methods assessing newborn characteristics using the New Ballard; gestational age as determined by LMP had a higher agreement with ultrasound determined gestational age and preterm birth. This finding is despite 18% of women being able to recall only the month of their LMP in the present study. The performance of LMP reported here was similar to that previously reported. LMP, on average, overestimated gestational age by ~1 day which is in the range of studies performed in other low- and middle-income countries–from an underestimation of 1 day to an overestimation of 4 days [10, 12, 13, 15].

While overall LMP appeared quite accurate, our present study demonstrated large and potentially clinically relevant variation in accuracy among individual participants with nearly half disagreeing by more than a week and wide 95% limits of agreement. This variation is greater than that seen in Brazil [14], Bangladesh [10] and Guatemala [13]. Two of these studies were prospective, routinely visiting households, recording LMP and actively recruiting newly pregnant women [10, 13]. The third study limited participation to women in the first trimester of pregnancy with prompts to facilitate recall, thus, potentially reducing recall bias [14]. A study where women similarly reported LMP at the first prenatal visit reported a similar variation in accuracy among participants [12]. We found that LMP overestimated gestational age when obtained early in pregnancy and underestimated gestational age when obtained later in pregnancy indicating a potential recall bias.

The accuracy of LMP was dependent on whether the infant was preterm or not; LMP consistently overestimated gestational age among preterm births more than term infants. However, another study using LMP found gestational age was underestimated in preterm infants compared to term infants [10]. That study also found significant differences in reporting accuracy based on maternal age and educational status; we did not find these differences in our study. This could potentially be due to the smaller sample size or very variation in educational achievement within our study population.

The wide variation in accuracy of gestational age estimates based on LMP led to a slight reduction in the proportion of preterm birth as compared to ultrasound and, more importantly, a low kappa statistic, sensitivity and specificity. LMP has previously been shown to lead to slight increases [10, 11, 14] and decreases [12, 13] in the proportion of preterm births. However, there was generally much stronger agreement between the methods than we witnessed [11, 16], even in low- and middle-income countries [10, 14]. These studies recruited participants very early in pregnancy [10, 11, 14], used very intensive LMP collection methods [10], or used calendars [13] and prompts [11, 14] to facilitate recall. Recruiting patients later in pregnancy in Pakistan without such methods reduced agreement, though LMP still performed better than reported here [12]. Thus, facilitating early access to prenatal care and using methods to facilitate recall may improve the accuracy of LMP estimates for use in low and middle income countries.

We found, as other studies have [7, 24], that the New Ballard overestimates gestational age compared to ultrasound. New Ballard was slightly more accurate among those with first trimester ultrasounds but still overestimated gestational age by 0.4 weeks, a similar magnitude difference to the -0.4 weeks found by Lee [23]. Consistent with work in Bangladesh [23], New Ballard underestimated gestational age among infants born small-for-gestational age in comparison to those of higher birthweights. This was the first study to note such a large overestimation of gestational age by New Ballard in preterm infants (nearly two weeks). This contributes to the marked reduction in the ability to detect preterm births using this method. However, sensitivity in our study was comparable to, and the specificity and diagnostic odds ratio were higher than, those seen in Bangladesh where New Ballard was performed by trained community health workers [23].

Most infants on whom the New Ballard was performed were between 35 and 39 weeks of gestational age at birth (62%) with most preterm births between 35 and 37 weeks (79%). Because physical characteristics and muscle tone are easier to differentiate at the extremes (very premature or post-mature), the New Ballard is less accurate between 32 and 41 weeks gestational age compared to 28 to 31 and greater than 42 weeks [24]. The range of gestational age estimates from New Ballard was smaller than that from ultrasound indicating infants being scored similarly despite varying gestational ages. While this effect was greater than that seen in a previous study in Guatemala [22], other studies have reported a similar narrowing of gestational age range when using the New Ballard [23]. Combining multiple elements into one score as is done with the New Ballard has been shown to make scoring more difficult [24], potentially accounting for smaller variance in score.

Given the degree of accuracy of the New Ballard within this study, its long training and test procedure and need for standardization, a shorter and more accurate alternative is desirable. One alternative is the 6-item Capurro, five of which were measured within this study as part of the New Ballard. Gestational age estimates by Capurro can be performed by trained birth attendants and have been shown to be highly correlated with those from New Ballard [22]. However, a similar study evaluating alternative methods of gestational age determination in rural Guatemala found that the Capurro performed similarly to the New Ballard results presented here (mean difference to ultrasound: -0.48 weeks for the Capurro versus +0.61 weeks in this study), with most preterm infants being misidentified as term [13]. Of particular note, Neufeld et al. used a form of the Capurro without any neuromuscular signs which we found were more strongly correlated with gestational age than physical signs. Additionally, in our study two of five scores used in the Capurro–ear and plantar creases—were not statistically significantly correlated with gestational age and only the breast score was significantly associated with prematurity.

A new alternative method can adapt the New Ballard or Dubowitz methods to find and use only those elements most highly correlated with gestational age, as in the development of the Capurro [20], or adjust the scores by birthweight [25, 26]. Only six of the individual elements of the New Ballard were associated with either gestational age or prematurity, the majority of which were neurological items. Of note, the only physical score significantly associated with preterm birth, breasts, was taught by assigning each number to the size of a food commonly eaten in the region (rice, lentil, bean, corn kernel). While neurological scores were previously found to correlate better with gestational age than physical scores [23], this pattern was reversed in other studies [24, 25]. Additionally, there were marked differences in correlations between individual elements across countries and studies [23–25]. However, those from Bangladesh agreed strongly with those reported here [23]. Additionally, other methods based on additional neonatal measurements can be developed [35, 36]. Given the varying levels of agreement across varying country and study contexts, any new methods must be developed and validated using a much larger and racially/ethnically diverse sample.

### Limitations

In Guatemala, few women have formal antenatal care during pregnancy and, thus, there may be a selection bias among study participants. The small sample size in this study limited the ability to look at differences in sensitivity and specificity for preterm birth among only those births with first trimester ultrasound and LMP reports. Additionally, results might not be generalizable to other contexts due to sample size. However, they fit with the broad trends from similar studies in low and middle-income countries [7, 12, 13, 24]. Ultrasound is most accurate earlier in pregnancies and, thus, would be the best standard by which to compare the other methods to determine gestational age. Ultrasounds for some women (n = 28) were not completed within 20 weeks, though, limiting the accuracy of the estimation for these women and potentially exacerbating differences among methodologies. The use of ultrasound as the standard requires the assumption that fetal growth in utero is consistent. However, fetal size references may fail to account for normal variability in fetal size [7] and assumes that gestational age is the only contributor [37]. Intrauterine growth restriction can begin as early as the first trimester of pregnancy though [38]. Additionally, New Ballard scores were converted to continuous gestational age estimates, rather than using the two week increments as published, which might have led to incorrect estimates.

## Conclusions

Preventing childhood mortality is essential for enhancing health security. Preterm birth is a major contributor to childhood mortality worldwide and identification of premature newborns is necessary for proper allocation of care and prevention of mortality. While prenatal ultrasound is the most accurate method for determining gestational age if performed early in pregnancy, it is frequently not available. LMP and New Ballard are widely available methods that have been shown to be quite accurate under ideal conditions. However, as presented here they suffered from wide variations in accuracy and had systematic biases based on varying newborn and maternal characteristics. Using these methods would have led to inaccurate estimations for rates of both preterm birth and small for gestational age. Given suboptimal performance, there is a need for simple, novel methods to accurately determine gestational age and preterm birth in low and middle-income countries where the burden is greatest, ultrasound is not readily available and women present late to antenatal care.

## Supporting information

S1 Dataset Dataset for the manuscript. (CSV)

S1 Fig Bland-Altman plots comparing gestational age estimates at birth by ultrasound to those from (A) the New Ballard newborn assessment and (B) LMP. Red dashed line indicates the mean difference between methods and blue lines indicate the upper and lower limits of agreement.
(PPTX)

S1 Table Gestational age estimates at birth within one week, ten days and two weeks of ultrasound for New Ballard and LMP. (DOCX)
