# Supplementary figures and images for: Determining gestational age and preterm birth in rural Guatemala: A comparison of methods
Source: PLoS One. 2018 Mar 19;13(3):e0193666. doi: 10.1371/journal.pone.0193666 (PMC5858755; doi:10.1371/journal.pone.0193666)

## Slide 1
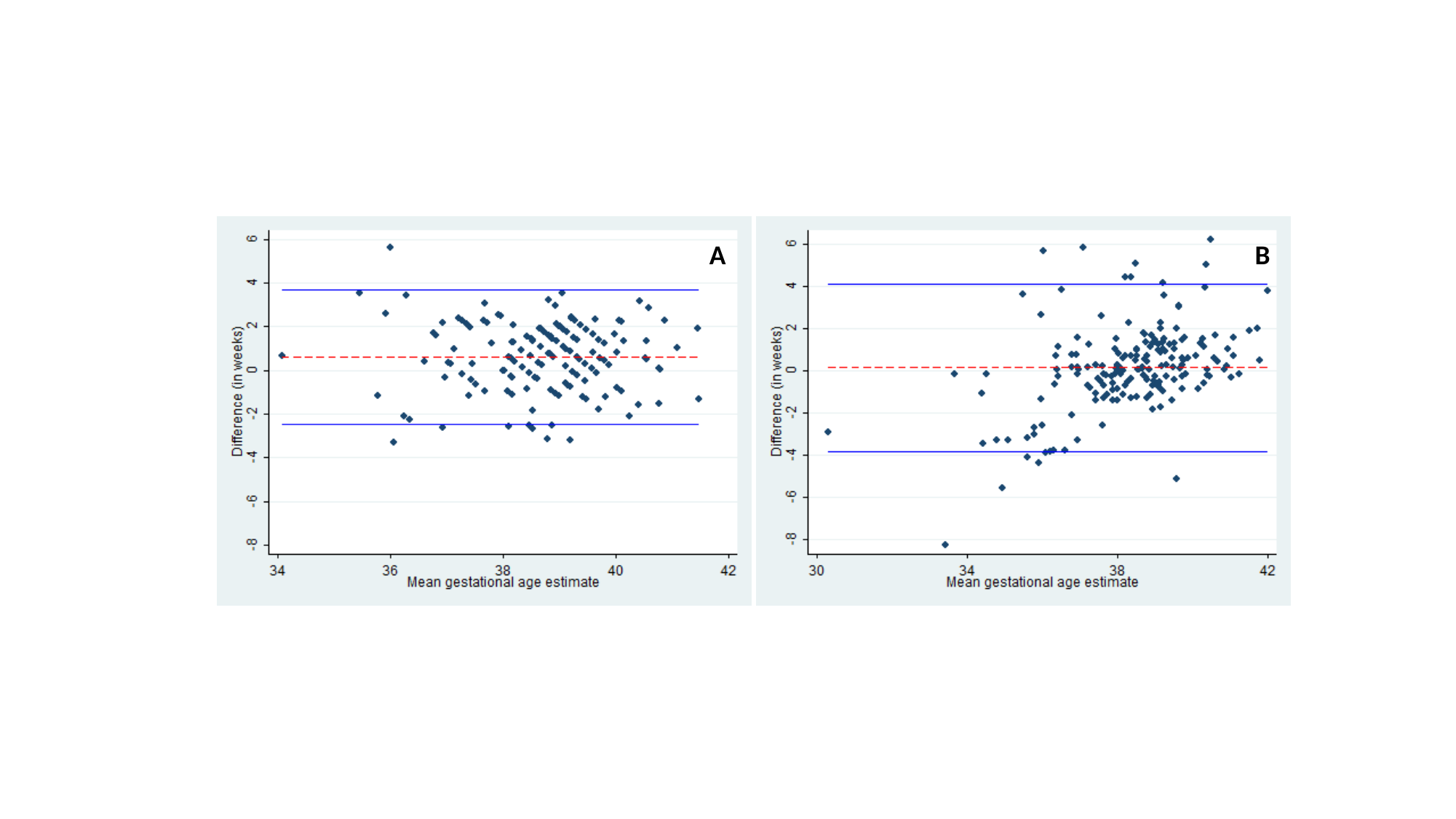

A
B

Supplement: S1 Fig — Red dashed line indicates the mean difference between methods and blue lines indicate the upper and lower limits of agreement. (PPTX) [file pone.0193666.s002.pptx]
