# Supplementary material for: Determining gestational age and preterm birth in rural Guatemala: A comparison of methods
Source: PLoS One. 2018 Mar 19;13(3):e0193666. doi: 10.1371/journal.pone.0193666 (PMC5858755; doi:10.1371/journal.pone.0193666)
Supplement: S1 Table — (DOCX) [file pone.0193666.s003.docx]

|  | **Difference from ultrasound** | | |
| --- | --- | --- | --- |
|  | **±7 days** | **±10 days** | **±14 days** |
| Ballard (n=156) | 65 (42%) | 89 (57%) | 116 (74%) |
| LMP (n=185) | 103 (56%) | 127 (69%) | 146 (79%) |
